# Cross-platform comparability of microarray technology: Intra-platform consistency and appropriate data analysis procedures are essential

**DOI:** 10.1186/1471-2105-6-S2-S12

**Published:** 2005-07-15

**Authors:** Leming Shi, Weida Tong, Hong Fang, Uwe Scherf, Jing Han, Raj K Puri, Felix W Frueh, Federico M Goodsaid, Lei Guo, Zhenqiang Su, Tao Han, James C Fuscoe, Z Alex Xu, Tucker A Patterson, Huixiao Hong, Qian Xie, Roger G Perkins, James J Chen, Daniel A Casciano

**Affiliations:** 1 National Center for Toxicological Research, U.S. Food and Drug Administration, 3900 NCTR Road, Jefferson, Arkansas 72079, USA; 2 Z-Tech Corporation, 3900 NCTR Road, Jefferson, Arkansas 72079, USA; 3 Center for Devices and Radiological Health, U.S. Food and Drug Administration, 2098 Gaither Road, Rockville, Maryland 20850, USA; 4 Center for Biologics Evaluation and Research, U.S. Food and Drug Administration, NIH Campus Building 29B, 29 Lincoln Drive, Bethesda, Maryland 20892, USA; 5 Center for Drug Evaluation and Research, U.S. Food and Drug Administration, 1451 Rockville Pike, Rockville, Maryland 20852, USA

## Abstract

**Background:**

The acceptance of microarray technology in regulatory decision-making is being challenged by the existence of various platforms and data analysis methods. A recent report (E. Marshall, *Science*, 306, 630–631, 2004), by extensively citing the study of Tan *et al*. (*Nucleic Acids Res*., 31, 5676–5684, 2003), portrays a disturbingly negative picture of the cross-platform comparability, and, hence, the reliability of microarray technology.

**Results:**

We reanalyzed Tan's dataset and found that the intra-platform consistency was low, indicating a problem in experimental procedures from which the dataset was generated. Furthermore, by using three gene selection methods (*i.e.*, *p*-value ranking, fold-change ranking, and Significance Analysis of Microarrays (SAM)) on the same dataset we found that *p*-value ranking (the method emphasized by Tan *et al*.) results in much lower cross-platform concordance compared to fold-change ranking or SAM. Therefore, the low cross-platform concordance reported in Tan's study appears to be mainly due to a combination of low intra-platform consistency and a poor choice of data analysis procedures, instead of inherent technical differences among different platforms, as suggested by Tan *et al*. and Marshall.

**Conclusion:**

Our results illustrate the importance of establishing calibrated RNA samples and reference datasets to objectively assess the performance of different microarray platforms and the proficiency of individual laboratories as well as the merits of various data analysis procedures. Thus, we are progressively coordinating the MAQC project, a community-wide effort for microarray quality control.

## Background

The U.S. Food and Drug Administration's (U.S. FDA) Critical Path white paper ) identifies pharmacogenomics and toxicogenomics as a promising tool in advancing medical product development and personalized medicine, and the guidance for the industry on pharmacogenomic data submissions has been released ). However, standardization is much needed before microarrays – a core technology in pharmacogenomics and toxicogenomics – can be reliably applied in clinical practice and regulatory decision-making [1-4]. Many commercial and in-house microarray platforms are in use, and a natural question is whether the results from different platforms are comparable and reliable [5]. As the U.S. FDA is actively assessing the applicability of microarrays as a tool in pharmacogenomic and toxicogenomic studies, we are particularly interested in information regarding the reliability of microarray results and the cross-platform comparability of microarray technology. Several studies that specifically address cross-platform comparability report mixed results [6-15]. Receiving particular attention is the Tan *et al*. study [11] which compares the results from three commercial platforms (Affymetrix, Agilent, and Amersham) and finds strikingly low cross-platform concordance, *i.e*., only four of the 185 unique genes identified as significantly up- or down-regulated by the three platforms are in common. The results of Tan's study are extensively cited in a recent report in *Science *[5] and quoted by other media (*e.g.*, ); they collectively portray a disturbingly negative picture regarding the cross-platform comparability and reliability of microarray technology.

The *Science *report [5] and the original article [11] appear to convey the message that the observed poor cross-platform concordance is largely due to inherent technical differences among the various microarray platforms. However, cross-platform comparability depends on intra-platform consistency that, unfortunately, is not sufficiently achieved and addressed in Tan's study [11]. As we know, many factors affect microarray data reproducibility and large differences in the quality of microarray data from different laboratories using the same platform exist [4,16]. Therefore, it is important not to confuse the poor performance obtained in a particular study with that achievable by the technology. We believe that appropriately assessing the reliability of microarray results and the cross-platform comparability of microarray technology is essential towards the proper use of microarray data and their acceptance in a regulatory setting.

Because Tan *et al*.'s paper [11] and the related *Science *report [5] have caused a lot of confusion to the microarray community, in this paper we set to closely re-examine the dataset of Tan *et al*. to determine the exact causes of the widely cited poor cross-platform concordance. We describe an alternative analysis of Tan's dataset with the intention to address several common issues related to cross-platform comparability studies such as intra-platform (technical and biological) consistency and the impact of different gene selection and data (noise) filtering procedures. We demonstrate that the main reason for the lack of concordance among the three platforms from Tan's study does not appear to be "because they were measuring different things" [5], but instead appears to be more likely because the original data [11] are of low intra-platform consistency and analyzed with a poor choice of methods. By analyzing the same dataset with a simple fold-change ranking and SAM (Significance Analysis of Microarrays) [17], we found a much higher cross-platform concordance than Tan *et al*.'s original analysis suggested.

We should point out that the purpose of our work is by no means a criticism of the study of Tan *et al*. In fact, the approach by which the data were analyzed by Tan *et al*. is *statistically *correct and widely used in microarray data analysis. The purpose of our work is to bring the issue on the assessment of the merits of statistical methods to the attention of statisticians and bioinformaticians while analyzing high-dimensional biological data such as microarray data [18-20]. Only after the validity of the data analysis methods is established can the biological significance of microarray results be reliably trusted.

Our results illustrate the need for establishing calibrated reference RNA samples and "gold standard" datasets (*e.g.*, by QRT-PCR) to objectively assess the performance of various platforms and individual microarray laboratories. Equally importantly, the merits of various data analysis procedures proposed for microarray data analysis must be rigorously assessed and validated before the regulatory utility of microarray data can be realized.

## Methods

### Dataset

The dataset, consisting of 2009 genes commonly tiled across the three platforms based on matching of GenBank accession numbers, is made publicly available by the original authors [11][21]. Briefly, differential gene expression in pancreatic PANC-1 cells grown in a serum-rich medium ("control" group) and 24 h following the removal of serum ("treatment" group) is measured using three commercial microarray platforms, *i.e*., Affymetrix (25-mer), Agilent (cDNA), and Amersham (30-mer) [11]. RNA is isolated from three control-treatment pairs of biological replicates (B1, B2, and B3) of independently cultured cells. For the first biological replicate pair (B1), the same RNA preparations are run in triplicates on each platform, resulting in three pairs of technical replicates (T1, T2, and T3) that only account for the variability of microarray technology. Therefore, for the one-color platforms (Affymetrix and Amersham), five hybridizations are conducted for the control samples and five hybridizations are done for the treatment samples. For the two-color platform (Agilent), dye-swap replicates are conducted, resulting in a total of 10 hybridizations. More details can be found in the original article [11].

For each platform, raw intensity data were logarithm (base 2) transformed and then averaged for genes with multiple representations on the microarray. The log ratio (LR) data were calculated based on the difference in log intensities (LI) between the two samples in a control-treatment pair. For the Affymetrix and Amersham platforms, the pairing of the control and treatment was conducted in such a way that it matched the pairing on the two-channel platform (Agilent). LR data for the dye-swap pair were averaged for the Agilent platform.

### Metrics for assessing data reproducibility

Data reproducibility was assessed according to three metrics, *i.e.*, log intensity correlation (LIr^2^), log ratio correlation (LRr^2^), and percentage of overlapping genes (POG), where r^2 ^is the squared Pearson correlation coefficient. POG represents the number of genes common in two or more "significant" gene lists (with consideration of regulation directionality) divided by L, the number of genes in a gene list. Unless indicated otherwise, in this study L was set to 100 (50 up and 50 down-regulated) so that the total number of unique genes (172) identified by our analysis from the three platforms is close to that (185) shown in the Venn diagram presented in the original article [11] and the report in *Science *[5].

### Data (noise) filtering

It has been suggested that expression data for genes marked with "present" (or of higher intensity) appear to be more reliable than those marked with "absent" (or of lower intensity) [9,13,22]. Without the "absent" call information from the dataset made available by Tan *et al*., we adopted a data filtering procedure proposed by Barczak *et al*. [9] by excluding 50% of the genes with the lowest average intensity across all hybridizations on each platform, resulting in a subset of 537 genes (out of 2009, *i.e*., 26.7%). This subset of 537 genes is presumably more reliably detectable on all the three platforms, whereas data points with lower intensity would more likely reflect platform-dependent noise structures or cross-hybridization patterns instead of real information of biological significance. The reduced subset of 537 genes was subjected to the same procedures for data quality assessment and gene selection.

### Gene selection methods

Three gene selection methods were applied for identifying differentially expressed genes between the two groups of samples: (i) fold-change ranking, (ii) *p*-value ranking, and (iii) SAM [17]. For fold-change ranking, LR data were rank-ordered and an equal number of genes (L, with each half from the up- or down-regulation direction) were selected from each of the platforms or replicates being compared in order to avoid ambiguity in calculating concordance. The method of fold-change ranking applies to situations where two or more replicates (or platforms) are being compared. However, both the *p*-value ranking and SAM methods are applicable where there is a sufficient number of replicates. In this study, both *p*-value ranking and SAM were only applied to select the same number of genes from each platform with the three biological replicate pairs (B1, B2, and B3), but not for the comparison of two replicate pairs. The *p*-values were calculated for each gene using a two-tailed Student's t-test. In practice, the ranking was performed based on the t-statistic, which carries the information regarding the direction (up or down) of regulation. Cross-platform concordance was measured as the overlap of genes identified from different platforms. Most discussions in this study were based on results from fold-change ranking with a selected number of genes L = 100 (50 up and 50 down) unless otherwise indicated. Different numbers of genes were also selected by the three gene selection methods.

## Results

### Intra-platform technical reproducibility

**Figure 1:**
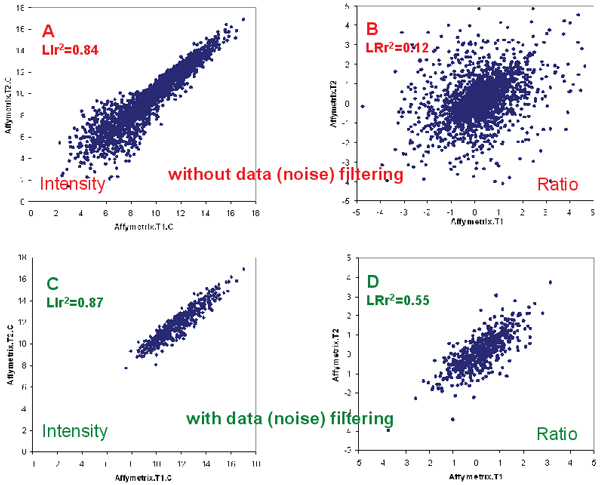
**Technical reproducibility. **A and C: The log2 intensity correlation of the control samples of technical replicate pairs T1 and T2 before (LIr^2 ^= 0.84) and after (LIr^2 ^= 0.87) data filtering, respectively; B and D: The log2 ratio correlation of the technical replicate pairs T1 and T2 before (LRr^2 ^= 0.12) and after (LRr^2 ^= 0.57) data filtering. Poor intra-platform consistency is more apparent in log ratios.

The intra-platform technical reproducibility can and should be high, but appears to be low in Tan's study [11], particularly for the Affymetrix platform. Specifically, intensity correlation of technical replicates for the Affymetrix data is low compared to data from others researchers [13,16,23] and our collaborators. A direct consequence of low LIr^2 ^(log intensity correlation squared) is very low LRr^2 ^(log ratio correlation squared): an average of 0.11 and 0.54 for before and after data filtering, respectively, corresponding to an average POG (percentage of overlapping genes) of 13% and 51% (based on the gene selection method of fold-change ranking), respectively (Tables 1 and 2). That is, when all 2009 genes are considered, only about 13% of the genes are expected to be in common between any two pairs of Affymetrix technical replicates, if 100 genes (50 up and 50 down) are selected from each replicate. In contrast, the percentage of commonly identified genes from two pairs of technical replicates is expected to be around 51% when the analysis is limited to the subset of 537 highly expressed genes. Figure 1 gives typical scatter plots showing the correlation of log intensity (Figures 1A and 1C) and log ratio (Figures 1B and 1D) data from the Affymetrix platform that indicate a low intra-platform consistency, especially before data filtering. The low intra-platform consistency is much more apparent for data in the log ratio space (Figures 1B and 1D). Since a primary purpose of a microarray gene expression study is to detect the difference in expression levels (*i.e*., fold-change or ratio), it is important to assess data consistency in the log ratio space (Figures 1B and 1D) in addition to in the log intensity space (Figures 1A and 1C).

**Figure 2:**
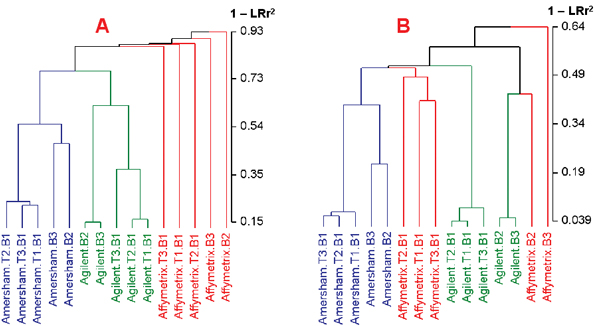
**Hierarchical clustering of replicate sample pairs. **Clustering was based on log ratios with average linkage and a distance metric of (1-LRr^2^), where LRr^2 ^is the squared Pearson correlation coefficient between the log ratios. The numbers represent (1-LRr^2^), which approximately equals the percentage of uncommon genes. A: Clustering based on the expression profiles across 2009 genes (without data filtering); B: Clustering based on the expression profiles across 537 genes (with data filtering). There is a dramatic increase in LRr^2 ^after filtering noisy data (note the different scales of the distance in each figure). Deficient technical and biological reproducibility on the Affymetrix platform from Tan's study [11] is evident. Technical reproducibility on the Agilent and Amersham platforms appears to be reasonable (B). However, although biological reproducibility can be high (*e.g.*, B2 and B3 on Agilent), there appears to be a clear separation of sample B1 from samples B2 and B3.

Technical reproducibility appears to be reasonable on the Amersham platform: average LRr^2 ^is 0.77 and 0.94 for the three pairs of technical replicates before and after data filtering, corresponding to a POG of 76% and 89%, respectively. For the Agilent platform, technical replicate pairs T1 and T2 appear to be very similar, but markedly different from T3 (Figure 2A). It is notable that the Cy5 intensities for a subset of spots with lower intensities for one hybridization of the dye-swap pair of T3 are significantly different from those of T1 and T2 (data not shown). The difference between T3 and T1 or T2 is much reduced after data filtering (Figure 2B), largely owing to the removal of the outlying lower intensity spots in T3. Overall, average LRr^2 ^on the Agilent platform is 0.70 and 0.94 for the three pairs of technical replicates before and after data filtering, corresponding to a POG of 62% and 84%, respectively.

It is evident from Figure 2 that intra-platform consistency of the Affymetrix data from Tan's study is much lower than that of the Amersham and Agilent platforms. A thorough evaluation of experimental procedures would be needed to better understand such poor performance of the Affymetrix platform from Tan's study.

### Intra-platform biological reproducibility

The intra-platform biological reproducibility appears to be low (Figures 2A and 2B, and Tables 1 and 2) for all three platforms. Biological replicate pairs B2 and B3 appear to be quite similar in the Agilent platform (with LRr^2 ^of 0.85 and 0.95, and POG of 73% and 85%, respectively, for before and after data filtering). B1, however, which is represented by the average of the three pairs of technical replicates (T1, T2, and T3), appears to be quite different from B2 and B3, with an average LRr^2 ^of 0.41 and 0.52, and POG of 37% and 49%, respectively, for before and after data filtering. The difference between B1 and B2 or B3 on the Amersham platform is also noticeable: with average LRr^2 ^of 0.49 and 0.61, and POG of 44% and 54%, respectively, for before and after data filtering; whereas B2 and B3 shows a higher LRr^2 ^of 0.53 and 0.78, and POG of 49% and 71% for before and after data filtering, respectively. Because of the low technical reproducibility of the Affymetrix data, it is not surprising that the biological reproducibility from the Affymetrix platform is low: with average LRr^2 ^of 0.10 and 0.45, and POG of 14% and 45% for before and after data filtering, respectively (Tables 1 and 2). One possible cause of the observed low biological reproducibility could be large experimental variations during the processes of cell culture and/or RNA sample preparation.

### Impact of data (noise) filtering

All 2009 genes, regardless of their signal reliability, are used in Tan's original analysis [11]. After adopting Barczak *et al*.'s data filtering procedure [9] by excluding 50% of the genes with the lowest average intensity on each platform, a subset of 537 genes having more reliable intensity measurement is obtained. As expected, a significant increase in both technical and biological reproducibility is observed (Figures 2A and 2B; notice the different scales shown in the distance metric). The impact of data filtering on data reproducibility is more apparent from Figures 1B and 1D when log ratios from technical replicate pairs T1 and T2 on the Affymetrix platform are compared. This simple data filtering procedure appears justifiable for cross-platform comparability studies, assuming that genes tiled on a microarray represent a random sampling of all the genes coded by a genome, and that only a (small) portion of the genes coded by the genome are expected to be expressed in a single cell type under any given biological condition; such is the case for the PANC-1 cells investigated in Tan's study [11].

Another subset consisting of 1472 genes that showed intensity above the median on at least one platform was subjected to the same analyses discussed for the datasets of 2009 and 537 genes. Gene identification was also conducted individually on each platform using the 50% of genes above the median average intensity, and the concordance was then compared using the three significant gene lists. In both cases, the identified cross-platform concordance was somewhere between that of the 2009-gene and 537-gene datasets (data not shown).

### Cross-platform comparability

**Figure 3:**
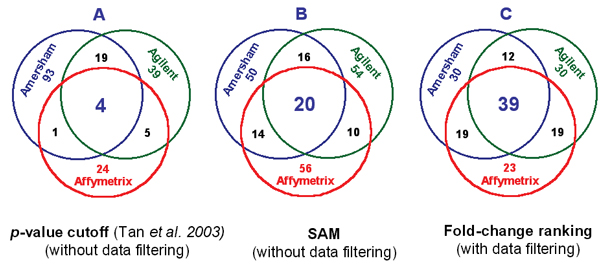
**Cross-platform concordance resulting from different data analysis procedures. **A: Poor cross-platform concordance (4/185) is reported [11] and cited [5]; B and C: Higher cross-platform concordance was observed by our analysis of the same dataset. For A, the number of selected genes from each platform is determined by the same statistical cutoff (alpha = 0.001), and the number of genes selected is 117, 77, and 34 for the Amersham, Agilent, and Affymetrix platforms, respectively. For B and C, the same number of genes (100) is selected from each platform by SAM (without data filtering) and fold-change ranking (with data filtering), respectively.

For each platform, the LR values of the three pairs of biological replicates (B1, B2, and B3) were averaged gene-wise and rank-ordered, and a list of 100 genes (50 up- and down-regulated) was identified. Without data filtering, 20 genes were identified to be in common by SAM (Figure 3B). With data filtering, 51 to 58 genes were found in common between any two platforms (Table 2), and 39 genes were in common to the three platforms, which identified a total of 172 unique genes (Figure 3C). While the overlap of 39 out of 172 is still low, the cross-platform concordance is some 10-fold higher than suggested by Tan's analysis (Figure 3A). The higher concordance reported here is a direct consequence of the data analysis procedure that incorporates filtering out genes of less reliability, selecting genes based on fold-change ranking rather than by a *p*-value cutoff, and selecting gene lists of equal length for each platform and for each regulation direction.

### Impact of gene selection methods on cross-platform comparability

As increasingly advanced statistical methods have been proposed for identifying differentially expressed genes, the validity and reliability of the more simple and "conventional" gene selection method by fold-change cutoff have been frequently questioned [24,25]. To compare the aforementioned results based on fold-change ranking with more *statistically *"valid" methods, we also applied SAM [17] and *p*-value ranking to the filtered subset of 537 genes to select 100 genes (50 up and 50 down-regulated) from the three pairs of biological replicates on each platform. For SAM, the POG between any two platforms ranged from 48% (Amersham-Agilent) to 58% (Affymetrix-Agilent), and 34 genes were found in common to the three platforms (Table 3). Of the 34 genes, 31 (91%) also appeared in the list of 39 genes selected solely based on fold-change ranking. Furthermore, 100 genes were also selected from each platform solely based on *p*-value ranking of the t-tests on the three pairs of biological replicate pairs, and 19 of them were found in common to the three platforms. Among the 19 genes, 11 (58%) appeared in the list of 39 genes selected by fold-change ranking.

However, when the three gene selection methods (*i.e*., *p*-value ranking, fold-change ranking, and SAM) were applied to the dataset of 2009 genes to select 100 genes from each platform (50 up and 50 down), much lower cross-platform concordance was obtained (Table 3): only 6, 14, and 20 genes were found in common to the three platforms by using *p*-value ranking, fold-change ranking, and SAM, respectively. The results indicate the importance of data (noise) filtering in microarray data analysis and the larger impact of the choice of gene selection methods on cross-platform concordance when the noise level is higher.

It is important to note that in both cases (2009-gene dataset and 537-gene dataset), *p*-value ranking yielded the lowest cross-platform concordance (Table 3). One explanation is that the *p*-value ranking method selected many genes with outstanding "statistical" significance but a very small fold change. Such a small fold change from one platform may be by chance or due to platform-dependent systematic noise structures (*e.g.*, hybridization patterns). Thus, such a small fold change is unlikely to be reliably detectable on other platforms, leading to low cross-platform concordance. For example, the gene (ID#1623) ranked as the most significant in up-regulation from the Affymetrix platform, exhibited a very "reproducible" log ratio measurement for the three biological replicate pairs (0.1620, 0.1624, and 0.1580, with a mean of 0.1608 and standard deviation of 0.002465). The *p*-value of the two-tailed Student t-test was 0.000078, representing the most statistically significant gene from the Affymetrix platform. However, the average log ratio of 0.1608 corresponds to a fold change of merely 1.12 (*i.e*., 12% increase in mRNA level). Such a small fold change is generally regarded as questionable by microarray technology currently available. On the Amersham platform, log ratios for the three replicates were -0.3648, 0.01624, and 0.04559, with a mean of -0.1010 (a fold change of -0.93, *i.e*., down-regulation by 7%), standard deviation of 0.2289, and *p *= 0.52. On the Agilent platform, log ratios for the three replicates were -0.1865, 0.2698, and 0.05786, with a mean of 0.04705 (a fold change of 1.03, *i.e*., up-regulation by 3%), standard deviation of 0.2283, and *p *= 0.75. In terms of *p*-value, this gene (ID#1623) was ranked as #1621 and #1785 out of 2009 genes on the Amersham and Agilent platforms, respectively; neither of these two platforms selected this gene as significant. When fold-change and SAM were applied for ranking genes based on the same Affymetrix data, the ranking of this gene was very low (ranked around #900 out of 2009 genes). Obviously, this gene was not selected by fold-change ranking owing to its small fold change (1.12).

Although fold-change ranking showed reasonable performance in terms of cross-platform concordance when applied to the subset of 537 genes, it is susceptible to selecting genes with a large fold change and large variability when the dataset is of low reproducibility, as is the case for the dataset with all 2009 genes. For example, one gene (ID#1245) was ranked as the 11^th ^largest fold change in up-regulation on the Affymetrix platform, but was only ranked in the top 500 and 120 by *p*-value ranking and SAM, respectively. The reason is that although this gene exhibited an average log ratio of 2.3432 (5.07-fold up-regulation), there was a large variability in the three biological replicate pairs (2.8986, 0.07195, and 4.0589), with a standard deviation of 2.058 and *p *= 0.19. The detected log ratios on the Amersham and Agilent platforms were 0.2955 (a fold change of 1.2273, *p *= 0.25) and 0.7566 (a fold change of 1.6895, *p *= 0.17), respectively, leading to a low ranking by both platforms either with fold-change ranking or *p*-value ranking.

SAM ranks genes based on a modified statistic similar to t-test: *delta *= *u*/(*s*+*s0*), where *u *stands for mean log ratio, *s *is defined as sqrt(*sd*^2^/*n*), and *n *is the number of replicates. By incorporating a fudge factor *s0 *in the denominator, in the calculation of *delta*, hence the ranking of genes, SAM effectively ranks genes relatively low in situations where either both *u *and *sd *are small, or when *u *and *sd *are both large [17]. Genes falling into these two situations will be ranked high by *p*-value ranking and fold-change ranking, respectively. Intuitively, SAM finds a tradeoff between fold-change and *p*-value, and should be regarded as a preferred gene selection method over pure *p*-value ranking or pure fold-change ranking.

It should be noted that many combinations of thorough statistical analyses and fold-change cutoff were conducted in Tan *et al*.'s original study [11]. However, the results that were emphasized and shown in the Venn diagram [5,11] (Figure 3A) are obtained from gene selection solely based on a statistical significance cutoff regardless of fold-change or signal reliability. Furthermore, because of the use of the same statistical significance cutoff, Tan's analysis resulted in an unequal number of selected genes from the three platforms and the two regulation directions. Therefore, the calculation of concordance becomes ambiguous and can underestimate cross-platform concordance.

### Results with different numbers of genes selected as significant

**Figure 4:**
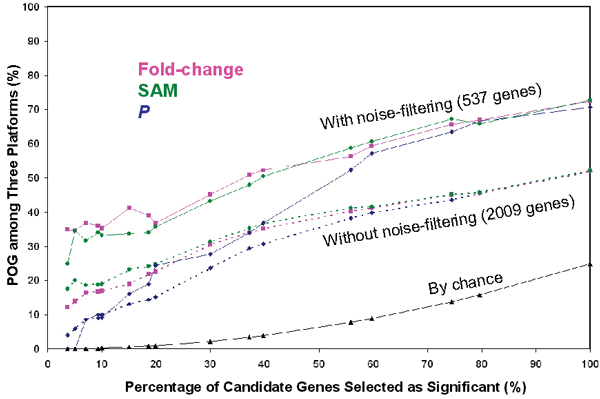
**POG at different percentages of genes selected as significant with three gene selection methods. **In both cases (with or without data filtering), *p*-value ranking resulted in much lower cross-platform concordance compared to fold-change and SAM, in particular when a small percentage (*e.g.*, <20%) of candidate genes are selected as significant, suggesting that the most significant genes selected by *p*-value ranking from one platform are unlikely to be selected as significant from another platform. POG by chance (assuming no correlation in log ratios, *i.e.*, LRr^2 ^= 0 among the three platforms) is also shown. See Figure 6 for more information on POG by chance.

In addition to selecting 100 genes (50 up and 50 down) from each platform (Table 3), different numbers of genes were selected by applying the three gene selection methods to both the 2009-gene and 537-gene datasets. The results are shown in Figure 4 and agree with the general conclusions discussed above when 100 genes were selected, *i.e*., data filtering increased cross-platform concordance and *p*-value ranking resulted in the lowest cross-platform concordance. Within the same dataset, the difference in POG by different gene selection methods diminishes as the percentage of selected genes increases. However, the POG difference due to gene selection methods is much more significant when the percentage of selected genes is small. The POG by *p*-value ranking is consistently lower than that by fold-change ranking or SAM. The extremely low POG when only a small percentage of genes are selected as significant indicates the danger of using *p*-value alone as the gene selection method.

Considering the large technical and biological variations identified in Tan's study, we conclude that the level of cross-platform concordance with the subset of 537 genes and by fold-change ranking or SAM is reasonable. Importantly, we observed no statistical difference between cross-platform LRr^2 ^and intra-platform biological LRr^2 ^after data filtering when all three platforms were considered (Table 2). However, it should be pointed out that the cross-platform LRr^2 ^was based on the correlation of the averaged log ratios over the three pairs of biological replicates from each platform as represented as Aff (Affymetrix), Ame (Amersham), and Agi (Agilent) in the right-bottom of Table 2.

### Relationship between LRr^2 ^and POG

**Figure 5:**
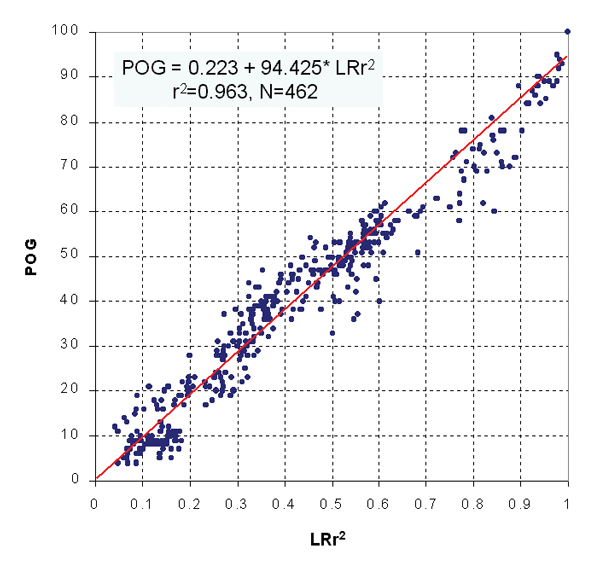
**Relationship between LRr2 and POG. **The squared pair-wise log ratio correlation (LRr^2^) and the percentage of overlapping genes (POG) showed a strong correlation (POG = 0.223 + 94.425* LRr^2^, r^2 ^= 0.963, N = 462). Each data point represents an LRr^2 ^~ POG pair from Tables 1 and 2.

From hundreds of pair-wise LRr^2 ^versus POG comparisons made on Tan's dataset (Tables 1 and 2), a strong positive correlation (r^2 ^= 0.963) between LRr^2 ^and POG (Figure 5) was observed. Therefore, it is essential to reach high log ratio correlation in order to achieve high concordance in cross-platform or intra-platform replicates comparisons.

### POG by chance

**Figure 6:**
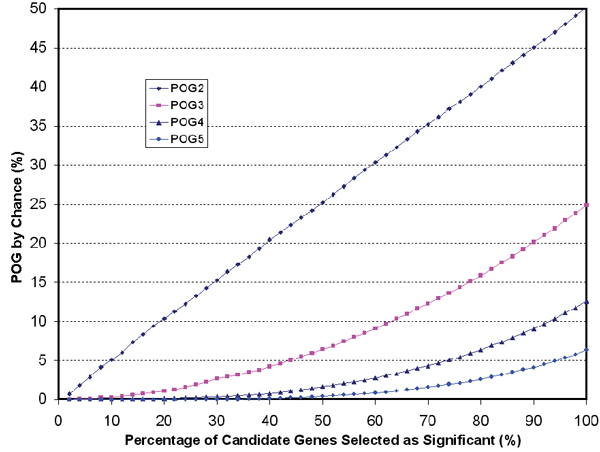
**POG by chance. **The percentage of overlapping genes (POG) increases by chance when the percentage (P) of selected genes (out of candidate genes) increases. For *k *replicates or platforms compared, the log ratios of the simulated replicates or platforms are assumed to have a normal distribution of *N*(0,1) and no correlation between each other (LRr^2 ^= 0). The expected POG by chance is 100*(P/2)^*k*-1^, where *k *is the number of replicates or platforms. POG2, POG3, POG4, and POG5 correspond to the comparison of 2, 3, 4, and 5 replicates (or platforms), respectively.

It should be noted that, in addition to cross-platform LRr^2^, POG also depends on the percentage P (between 0 and 1) of the total number of candidate genes selected as "significant". As an illustration, Figure 6 shows simulated POG results from random data of normal distribution of *N*(0,1), where there is no correlation between replicates or platforms (*i.e*., LRr^2 ^= 0). For the comparison of two replicates or platforms, a POG of 100*(P/2) is expected by chance and the other 100*(P/2) is expected to be dis-concordant in the directionality of regulation. For example, if all genes (P = 100%) are "selected" as significant (50% up and the other 50% down) for both replicates or platforms, by chance one would expect 50% of the total number of selected genes to be concordant in regulation direction (the other 50% of selected genes will be in opposite directions). For the comparison of three replicates or platforms, the percentage of genes expected to be concordant by chance is 100*(P/2)^2^; therefore, 25% of genes are expected to be concordant if all genes are "selected". For the comparison of *k *platforms (or replicates), the POG expected by chance would be 100*(P/2)^*k*-1^. The POG by chance is independent on the choice of gene selection methods.

## Discussion

We analyzed the dataset of Tan *et al*. [11] using an alternative approach and illustrated a number of unaddressed issues of their study. Briefly, Tan *et al*.'s study suffered from low intra-platform consistency and poor choice of data analysis procedures. Our analysis reiterates the importance of data quality assessment and the need for guidelines on data analysis. The impact of data (noise) filtering in microarray data analysis is shown and the problem of using *p*-value ranking as the only criterion for gene selection is highlighted. For microarray studies including cross-platform comparisons, it is essential to ensure intra-platform consistency by using appropriate quality control metrics and thresholds against the performance achievable on each platform.

Our data analysis procedures first involved a data (noise) filtering procedure that excludes 50% of the genes with the lowest average intensity on each platform. Secondly, an equal number of differentially expressed genes were selected from each platform, with half from up- and half from down-regulation, in order to avoid ambiguity in the calculation of concordance. Notice that the number of genes identified as up- and/or down-regulated depends on many factors such as the intrinsic nature of the biological samples, the number of gene probes present on the platform, the reproducibility (precision) of the platform, and the cutoff value of significance level. Therefore, the number of genes to be identified from each platform in a given study could be arbitrary, but in practice is limited by the number of genes that the biologist is interested in or is capable of examining in greater detail. It should be noted that for platforms with different reproducibility, the *p*-value or false discovery rate (FDR) cutoff will most likely be different when the same number of genes are selected based on fold-change ranking. However, for dataset of reasonable consistency, most genes selected by fold-change ranking also pass a *p*-value cutoff. Alternatively, when the same statistical cutoff (*e.g.*, a *p*-value < 0.001) is applied to different platforms, a platform that demonstrates higher consistency will select more genes than that with lower consistency, as shown in Figure 3A. Thirdly, we compared three different gene selection methods (*p*-value ranking, fold-change ranking, and SAM) and compared the cross-platform concordance. The results illustrate the danger of solely using *p*-value ranking in gene selection without considering fold change. On the other hand, fold-change ranking appears to perform well in identifying gene lists with large cross-platform overlap, which is a reasonable surrogate for assessing the accuracy of microarray data [14]. The most reliable results should be those genes showing low *p*-value and large fold change.

Overall, based on the same dataset of Tan *et al*., our reanalysis gives a cross-platform concordance (39 out of 172) some 10-fold higher than reported by the original authors [11] and extensively cited in *Science *[5], where only 4 out of 185 genes are found in common. Due to the limited quality of the dataset of Tan *et al*., it is reasonable to expect a higher cross-platform concordance when the quality of data from each platform increases to the best achievable levels. Reasonable cross-platform concordance can and should be attainable if microarray experiments are conducted at the level of performance achievable by the technology and if the resulting data are analyzed with validated methods.

It should be noted that POG depends on the percentage (P) of genes selected out of the candidates; the higher percentage selected the higher the POG (Figure 4). When the results identified from the dataset of 2009 genes were compared to those from the subset of 537 genes, the results were based on the selection of the same number of 100 genes (50 up and 50 down) from each platform, corresponding to 4.98% and 18.62%, respectively, out of the total numbers of the candidate genes. The corresponding percentages of concordant genes by chance for the comparison of any two platforms are 2.49% and 9.31%. For the comparison of three platforms, the corresponding percentages of overlap by chance are approximately 0.25% and 0.87% for the 2009-gene dataset and 537-gene subset, respectively. Therefore, such a bias of POG towards a higher percentage of selected genes should be kept in mind when reading the numbers from comparing the two datasets, especially when two platforms are compared.

Increasingly complicated statistical methods have been continually proposed for identifying differentially expressed genes, and the validity and reliability of the simple gene selection method by fold-change ranking (cutoff) have been questioned [24,25]. The preference of using a statistical significance metric (*e.g*., *p*-value) as the gene selection method [25] is biased to random noise and platform-dependent systematic errors, resulting in the selection of genes with tiny fold changes that are indiscernible by currently available microarray technology. The fact that fold-change ranking identified a much higher percentage of concordant genes among the three platforms than *p*-value ranking is not difficult to understand when we consider microarray as a measurement tool and its fluorescence intensity detection is subject to various sources of variability. Therefore, only those fold changes that are above random intensity variation are reliable.

### Multi-factorial nature of cross-platform concordance

One of the goals of gene expression studies is to reliably identify a subset of genes that are differentially expressed in two different types of samples. Our results (Figure 5) demonstrate that it is essential to reach high log ratio correlation (LRr^2^) between two replicates or platforms in order to achieve high consistency between the lists of identified genes. There are several ways to increase LRr^2 ^and the most important steps should be setting quality control checkpoints to make sure that experimental variability is kept as small as possible so that, in turn, data from the same platform are reliable. After data collection, a reasonable data (noise) filtering procedure should be applied to exclude a portion of genes with the lowest intensity that likely reflects platform-specific noise structures (*e.g.*, cross-hybridization patterns). Increasing the number of replicates is theoretically important, but in practice is limited by the available resources. It is worth noting that the log ratio correlation of replicates largely depends on the magnitude of true biological differences in expression levels between the two groups of samples compared. For the comparison of dramatically different types of samples (*e.g.*, two different types of tissues or cell lines), the expected fold change for many genes is large, resulting in reproducibly measurable fold change for many genes. On the other hand, when the inherent biological differences between the two groups of samples are small (*e.g.*, control animals versus animals chronically treated with a chemical in low-dose, or two truly different cell populations that are "diluted" with common, unchanged, larger cell populations as seen in neurotoxicological studies), the reproducibility of the measured fold change is expected to be lower. For the detection of such subtle changes in gene expression, it is essential to optimize microarray protocols to obtain the best performance that is achievable.

### Inherent differences among various platforms

Our analysis amplifies the need for appropriate metrics and thresholds to objectively assess the quality of microarray data prior to devoting effort to more advanced statistical analysis. Our work also reiterates the urgent need for guidance on consistency in analyzing microarray data [4,26,27]. We agree that inherent technical differences among various microarray platforms exist because of differences such as probe length and design, patterns of cross-hybridization and noise structures, as well as experimental protocols. For example, the intra-platform consistency for the Amersham and Agilent platforms is significantly higher, but the concordance between these two platforms was not higher than the cross-platform concordance involving the Affymetrix platform (Figure 3B), which showed the lowest intra-platform consistency. In addition, as shown in Table 2, the three technical replicate pairs (T1, T2, and T3) on both Agilent and Amersham platforms showed the same average LRr^2 ^of 0.94 and an average POG of 84% (Agilent) and 89% (Amersham), but the cross-platform LRr^2 ^(between Agi.B1 and Ame.B1) was only 0.60, corresponding to a POG of 61%. Such a difference (LRr^2 ^of 0.94 versus 0.60, and POG of 84%/89% versus 61%) could be a result of inherent platform differences, *e.g*., cross-hybridization patterns due to differences in probes (cDNA versus 30-mer), and differences in detection methods (two-channel versus one-channel). The "true" cross-platform differences, *e.g.*, whether the probes from different platforms supposedly measuring the same gene are in fact targeting different regions or splicing variants of the same gene [13,28], should be resolved with more reliable datasets. The lack of gene identity information from the dataset made public by Tan *et al*. prevented us from using probe sequence matching to determine gene overlap across different platforms [28] and to assess the degree of improvement of cross-platform concordance.

### Calibrated reference RNA samples and "gold standard" datasets

Because the U.S. FDA is expected to receive microarray-based pharmacogenomic data as part of product submissions from the industry, data quality is of great concern. Although cross-platform concordance is important, what is more important is the accuracy of each platform. However, the accuracy of microarray technology has not been extensively assessed due to the lack of calibrated reference RNA samples and "gold standard" measurements. We are coordinating the MAQC (Microarray Quality Control) project [29] ( or ) aimed at assessing the performance achievable on various microarray platforms through a collaborative effort among six FDA Centers, the National Institute of Standards and Technology (NIST), the U.S. Environmental Protection Agency (EPA), major microarray platform providers (*e.g*., Affymetrix, Agilent, Applied Biosystems, GE Healthcare and Illumina), RNA sample providers (*e.g*., Ambion, Clontech and Stratagene), selected microarray users (*e.g.*, NCI, UCLA and UMass), and other stakeholders. Reference datasets will be generated on a pair of readily accessible RNA samples for each species (human, mouse, and rat) by multiple laboratories using multiple platforms, and will be made publicly available for objective assessment of intra-platform consistency, cross-platform comparability, and the comparison of various data analysis methods. Importantly, the relative expression levels for over one thousand genes in these samples will be measured by QRT-PCR and other independent technologies. The resulting "gold standard" datasets will be used to assess the accuracy of various microarray platforms. We expect that the "calibrated" reference RNA samples, reference datasets, and the resulting quality control metrics and thresholds will facilitate regulatory review of genomic datasets. Individual microarray laboratories can optimize and standardize their SOPs by using the same pair of RNA samples and checking their data quality against the reference datasets. By using these tools, a procedural failure may be identified and corrected, and the intrinsic technical differences among platforms can be better understood and addressed. The MAQC project, which is highly complementary to on-going efforts of the External RNA Controls Consortium (ERCC, ) and NIST's Metrology for Gene Expression Program ), will help move the process of standardizing microarray technology one step further.

### Quality control metrics and thresholds

Quality control metrics (parameters) need to be established for assessing the quality of microarray data. Equally important, thresholds for the quality control parameters should be established to determine whether the data quality from a study is acceptable. Before any advanced statistical analysis, exploratory analysis of microarray data in terms of the quality metrics (*e.g.*, LIr^2^, LRr^2^, and POG) may be used to identify irregularities in the data. The reference RNA samples and the reference datasets mentioned above will be essential to determine quality control thresholds.

### The need of guidance for microarray data analysis

Guidance on data analysis is needed in the standardization of microarray technology. A significant portion of the more than 10000 literature references on microarrays [4,18] deals with various strategies on data analysis. However, many of the methods or procedures have not been independently validated for their merits and limitations [18,30]. It is expected that reference datasets will enable a more reliable assessment of the merits of various procedures and methods for microarray data analysis. It is important not to compromise accuracy for the sake of reproducibility in microarray data analysis [31]. Unfortunately, many methods (*e.g.*, *p*-value or FDR cutoff) currently used in microarray data analysis appear to focus on reproducibility because of the lack of independent datasets for cross-validation. With the availability of "gold standard" measurements and cross-platform datasets from the calibrated reference RNA samples, it is possible to judge the performance of individual data analysis methods against the "true" values, not against themselves (*i.e*., data from the same platform in the same study).

We realize that the absence of control (comparison) data, *e.g. *from QRT-PCR analysis, limits the conclusions that can be drawn from Tan's dataset. Ultimately, it is the accuracy of the platform that determines its usefulness in research. It is also possible that different data analyses need to be used for specific platforms. As already indicated in the "note added in proof" of Tan *et al*. [11], a comparison between the Affymetrix platform and a long-oligo platform have revealed high concordance when used with identical RNA preparations [9]. However, before QRT-PCR data become available for a large subset of genes for the same pair of reference RNA samples, we suggest the use of cross-platform concordance as a surrogate of accuracy in order to evaluate the performance of different data analysis methods. Preliminary results illustrated in this paper indicate the limitations of *p*-value ranking (or *p*-value cutoff) when used alone as the gene selection method. The reliability of gene selection based on fold-change ranking has been demonstrated for datasets of higher quality when compared to the results from more sophisticated SAM method.

## Conclusion

Our reanalysis of the dataset of Tan *et al*. [11] illustrates two paramount challenges facing the microarray community. The first challenge is to ensure that individual microarray laboratories perform the bench work in a proficiency that is achievable by the technology. The second challenge is to critically evaluate and validate the merits of various data analysis methods (procedures). Currently, there is a lack of appropriate tools for microarray users to objectively assess the performance of microarray laboratories. In addition, as a community, we are not in short of "novel" methods for analyzing microarray data; on the contrary, the user is being faced with too many options and the true merits of such methods (procedures) have not been adequately evaluated. The outcomes of the ERCC and MAQC efforts will greatly help address the two challenges facing the microarray community, leading to more reliable, wider applications of the microarray technology.

## Abbreviations

LIr^2^: squared log intensity correlation coefficient; LRr^2^: squared log ratio correlation coefficient; POG: percentage of overlapping genes; SAM: Significance analysis of microarrays.

## Authors' contributions

LS had the original idea on the method and performed all data analysis and simulations, and wrote the manuscript. WT, HF, US, JH, ZS, HH and QX were involved in discussions on the data analysis and verified some of the calculations. JCC provided advices in statistics and suggested the presentation of results shown in Table 3 and Figure 4. JH, RKP, FWF, FMG, LG, TH, JCF, ZAX, TAP, RGP, JCC and DAC provided additional insights regarding issues on cross-platform comparison and microarray quality control. WT, RKP, JH, LG, JCF, RGP, JJC and DAC assisted with writing the manuscript. All authors participated in the design of the study and approved the final manuscript.

## Figures and Tables

**Table 1:** Data consistency for the dataset of 2009 genes (before data filtering). The pair-wise log ratio correlation squared (LRr^2^, lower triangle) and the percentage of overlapping genes (POG, upper triangle) are listed. T1, T2, and T3 are technical replicates; B1, B2, and B3 are biological replicates. The last three rows/columns (Affymetrix (Aff), Amersham (Ame), and Agilent (Agi)) represent results from the average of the three biological replicates. Gene selection was based on fold-change ranking, and a total of 100 genes (50 genes from each regulation direction) were selected for each comparison. Black bold numbers represent intra-platform technical or biological consistency, or cross-platform concordance.

	**Aff.T1**	**Aff.T2**	**Aff.T3**	**Ame.T1**	**Ame.T2**	**Ame.T3**	**Agi.T1**	**Agi.T2**	**Agi.T3**	**Aff.B1**	**Aff.B2**	**Aff.B3**	**Ame.B1**	**Ame.B2**	**Ame.B3**	**Agi.B1**	**Agi.B2**	**Agi.B3**	**Aff**	**Ame**	**Agi**
**Aff.T1**	Aff.T1	**18**	**10**	11	11	10	9	8	9	42	11	13	11	8	6	8	7	10	23	9	9
**Aff.T2**	**0.1229**	Aff.T2	**11**	7	9	8	5	7	6	36	19	15	9	10	9	5	6	7	23	9	8
**Aff.T3**	**0.1112**	**0.0893**	Aff.T3	10	7	10	11	10	11	37	12	10	9	8	7	11	9	9	22	8	12
**Ame.T1**	0.1548	0.1425	0.1587	Ame.T1	**72**	**78**	31	31	28	24	5	9	84	49	42	32	23	22	21	73	29
**Ame.T2**	0.1656	0.1701	0.1771	**0.7562**	Ame.T2	**78**	35	34	33	22	5	8	84	40	34	36	19	22	18	63	31
**Ame.T3**	0.1536	0.1521	0.1705	**0.7821**	**0.7743**	Ame.T3	28	28	28	23	5	9	87	43	38	29	19	21	20	67	28
**Agi.T1**	0.1307	0.1364	0.1732	0.3176	0.3325	0.3213	Agi.T1	**81**	**53**	19	5	9	33	20	17	88	38	38	22	29	63
**Agi.T2**	0.1453	0.161	0.1674	0.3281	0.3499	0.3362	**0.839**	Agi.T2	**51**	19	6	8	32	21	18	86	36	38	23	30	61
**Agi.T3**	0.114	0.1358	0.1369	0.258	0.2867	0.2637	**0.5895**	**0.6818**	Agi.T3	17	4	4	31	21	16	60	31	29	17	28	45
**Aff.B1**	0.5515	0.5478	0.5551	0.2755	0.3102	0.288	0.2661	0.2866	0.234	Aff.B1	**16**	**13**	25	18	16	20	17	19	40	24	21
**Aff.B2**	0.048	0.0877	0.0422	0.0612	0.0603	0.0664	0.0595	0.0681	0.047	**0.1045**	Aff.B2	**14**	4	7	9	5	9	9	33	6	10
**Aff.B3**	0.0667	0.0852	0.076	0.1191	0.111	0.1255	0.1132	0.1224	0.0856	**0.1377**	**0.061**	Aff.B3	8	6	8	9	7	9	40	9	12
**Ame.B1**	0.1719	0.1684	0.1836	0.9171	0.9144	0.9245	0.3525	0.368	0.2932	0.3168	0.0682	0.129	Ame.B1	**48**	**40**	34	21	23	20	73	31
**Ame.B2**	0.1059	0.0963	0.0801	0.5138	0.5063	0.4695	0.1445	0.1557	0.1145	0.1694	0.0754	0.0956	**0.5403**	Ame.B2	**49**	21	30	27	20	62	27
**Ame.B3**	0.0871	0.0835	0.0677	0.4045	0.3361	0.4771	0.1301	0.1396	0.0864	0.1431	0.0734	0.116	**0.4397**	**0.529**	Ame.B3	16	30	34	20	58	28
**Agi.B1**	0.1451	0.1614	0.178	0.3364	0.3613	0.3429	0.8965	0.9314	0.8457	0.2932	0.0646	0.1189	0.3775	0.1539	0.131	Agi.B1	**37**	**38**	23	29	62
**Agi.B2**	0.0969	0.0883	0.0912	0.1982	0.176	0.1955	0.3946	0.4142	0.2584	0.1668	0.1263	0.103	0.2066	0.2835	0.3004	**0.3926**	Agi.B2	**73**	21	30	69
**Agi.B3**	0.1105	0.094	0.0984	0.1985	0.1846	0.1944	0.4178	0.4358	0.3152	0.1825	0.1251	0.1221	0.2095	0.2699	0.3055	**0.4334**	**0.8483**	Agi.B3	24	33	70
**Aff**	0.266	0.3221	0.2698	0.2417	0.2466	0.2555	0.2324	0.2542	0.1889	0.5176	0.5015	0.6006	0.2699	0.1957	0.1976	0.2508	0.2346	0.2555	Aff	**24**	**27**
**Ame**	0.1522	0.1452	0.1364	0.7625	0.7245	0.78	0.2579	0.2733	0.2008	0.2616	0.0893	0.1419	0.8224	0.8208	0.771	0.2716	0.3197	0.3181	**0.2772**	Ame	**36**
**Agi**	0.1477	0.145	0.1552	0.3087	0.3047	0.3087	0.7209	0.7515	0.5939	0.2708	0.1204	0.1418	0.3346	0.2721	0.2744	0.7693	0.8285	0.8607	**0.3045**	**0.3674**	Agi

**Table 2:** Data consistency for the dataset of 537 genes (after data filtering). The pair-wise log ratio correlation squared (LRr^2^, lower triangle) and the percentage of overlapping genes (POG, upper triangle) are listed. T1, T2, and T3 are technical replicates; B1, B2, and B3 are biological replicates. The last three rows/columns (Affymetrix (Aff), Amersham (Ame), and Agilent (Agi)) represent results from the average of the three biological replicates. Gene selection was based on fold-change ranking, and a total of 100 genes (50 genes from each regulation direction) were selected for each comparison. Black bold numbers represent intra-platform technical or biological consistency, or cross-platform concordance.

	**Aff.T1**	**Aff.T2**	**Aff.T3**	**Ame.T1**	**Ame.T2**	**Ame.T3**	**Agi.T1**	**Agi.T2**	**Agi.T3**	**Aff.B1**	**Aff.B2**	**Aff.B3**	**Ame.B1**	**Ame.B2**	**Ame.B3**	**Agi.B1**	**Agi.B2**	**Agi.B3**	**Aff**	**Ame**	**Agi**
**Aff.T1**	Aff.T1	**53**	**56**	52	49	52	50	53	54	71	46	41	53	42	46	52	45	44	58	52	52
**Aff.T2**	**0.5745**	Aff.T2	**44**	51	53	51	48	47	46	70	46	45	53	41	41	47	43	43	61	48	47
**Aff.T3**	**0.5902**	**0.4525**	Aff.T3	52	51	52	53	54	53	69	37	37	53	36	39	53	38	37	48	46	47
**Ame.T1**	0.5341	0.53	0.572	Ame.T1	**88**	**90**	58	60	54	60	39	38	95	55	52	60	37	38	53	76	50
**Ame.T2**	0.5439	0.537	0.5836	**0.9347**	Ame.T2	**89**	56	58	52	59	40	39	92	57	51	58	36	36	55	77	48
**Ame.T3**	0.5486	0.5499	0.5768	**0.9374**	**0.9482**	Ame.T3	57	59	53	59	40	40	94	55	54	58	36	37	55	78	48
**Agi.T1**	0.4382	0.446	0.5163	0.564	0.5705	0.572	Agi.T1	**89**	**78**	57	33	40	59	35	37	89	44	50	50	49	69
**Agi.T2**	0.4676	0.4847	0.5405	0.5954	0.6009	0.6052	**0.9613**	Agi.T2	**84**	57	38	42	62	39	39	93	49	55	53	50	75
**Agi.T3**	0.4553	0.4617	0.5177	0.5687	0.5729	0.5796	**0.9041**	**0.9419**	Agi.T3	56	38	40	55	36	38	88	47	52	50	46	74
**Aff.B1**	0.858	0.8009	0.8076	0.664	0.6755	0.68	0.5682	0.6059	0.5822	Aff.B1	**41**	**44**	60	44	47	57	45	47	64	55	55
**Aff.B2**	0.4191	0.4626	0.361	0.3498	0.3524	0.3606	0.3054	0.334	0.3301	**0.5029**	Aff.B2	**41**	41	47	53	36	54	52	58	46	47
**Aff.B3**	0.3238	0.417	0.2726	0.3576	0.3766	0.3844	0.3515	0.3834	0.3774	**0.4083**	**0.4451**	Aff.B3	40	38	43	41	44	47	74	46	48
**Ame.B1**	0.5534	0.5502	0.5894	0.9767	0.9807	0.9818	0.5806	0.6129	0.5856	0.6871	0.3616	0.3806	Ame.B1	**56**	**53**	61	37	38	55	78	50
**Ame.B2**	0.4268	0.3686	0.3707	0.6163	0.6253	0.6093	0.313	0.3422	0.3289	0.4719	0.4917	0.2973	**0.6297**	Ame.B2	**71**	37	48	46	48	72	43
**Ame.B3**	0.4137	0.371	0.3575	0.579	0.5647	0.6168	0.3084	0.3389	0.3244	0.4624	0.5056	0.3427	**0.5989**	**0.7859**	Ame.B3	37	55	53	52	70	50
**Agi.B1**	0.4634	0.4739	0.5363	0.5885	0.594	0.5982	0.977	0.9889	0.9686	0.5979	0.3298	0.3784	0.6059	0.3348	0.3306	Agi.B1	**47**	**52**	51	50	74
**Agi.B2**	0.3926	0.3393	0.3296	0.3591	0.3581	0.3627	0.4694	0.5186	0.5031	0.4293	0.5678	0.3239	0.3674	0.5783	0.5682	**0.5072**	Agi.B2	**85**	53	45	72
**Agi.B3**	0.3888	0.3461	0.332	0.355	0.359	0.3598	0.4975	0.541	0.5335	0.4318	0.5659	0.353	0.3654	0.5435	0.5677	**0.5349**	**0.9525**	Agi.B3	53	46	78
**Aff**	0.6397	0.694	0.5684	0.5686	0.5842	0.5934	0.5141	0.5563	0.5432	0.7705	0.7688	0.7978	0.5942	0.5167	0.5437	0.5491	0.5396	0.5566	**Aff**	**58**	**58**
**Ame**	0.5379	0.4995	0.5108	0.842	0.8412	0.856	0.4661	0.5019	0.4805	0.6277	0.5041	0.3921	0.8639	0.8909	0.8765	0.4931	0.5547	0.5425	**0.6341**	**Ame**	**51**
**Agi**	0.4877	0.4581	0.4759	0.5176	0.5212	0.5249	0.7747	0.816	0.7989	0.5766	0.5354	0.4123	0.532	0.5341	0.5366	0.8137	0.8863	0.9037	**0.6372**	**0.6098**	**Agi**

**Table 3:** Percentage of overlapping genes (POG) determined by three gene selection methods. For each gene selection method, different percentages of genes (P) are selected from each platform.

Percentage (P, %)	2009-gene Dataset				537-gene Dataset				POG by chance
			
	Number of genes	*p*-value	Fold	SAM	Number of genes	*p*-value	Fold	SAM	
3.68	74	4.0	12.2	17.6	20	0	35.0	25.0	0.034
4.98	**100**	**6.0**	**14.0**	**20.0**	27	0	34.6	34.6	0.062
7.02	141	8.6	16.4	18.6	38	8.6	36.8	31.6	0.12
9.31	187	9.1	16.7	18.8	50	10.0	36.0	34.0	0.22
9.96	200	10.0	17.0	19.0	54	9.2	35.2	33.3	0.25
14.98	301	13.2	18.9	23.2	81	16.2	41.2	33.7	0.56
18.62	374	14.4	21.9	24.1	**100**	**19.0**	**39.0**	**34.0**	0.87
19.91	400	15.2	22.5	24.7	107	24.5	36.8	35.8	0.99
30.01	603	23.6	30.6	31.4	161	27.8	45.1	43.2	2.25
37.18	747	29.4	34.0	35.2	200	34.0	51.0	48.0	3.46
39.82	800	30.7	35.1	36.6	214	36.9	52.3	50.5	3.96
55.90	1123	38.3	40.3	41.2	300	52.3	56.3	58.7	7.81
59.73	1200	39.8	41.2	41.5	321	57.2	59.4	60.6	8.92
74.51	1497	43.5	45.2	45.1	400	63.5	65.7	67.2	13.88
79.64	1600	45.4	45.6	45.7	427	66.6	67.0	65.9	15.86
100.00	2009	51.9	52.0	52.2	537	70.7	72.4	72.8	25.00
